# Evaluation of a novel simulation-based training for urgent laryngectomy care

**DOI:** 10.1186/s12909-025-06964-8

**Published:** 2025-03-26

**Authors:** Freya Sparks, Nicky Gilbody, Katerina Hilari

**Affiliations:** 1 Present Address: Division of Language and Communication Science, City St Georges, University of London, 1 Myddelton Street, London, EC1R 1UW UK; 2 Speech and Language Therapy Department, Barts Health NHS Trust, The Royal London Hospital, Whitechapel, London, E1 1FR UK

**Keywords:** Laryngectomy, Simulation, Skill development, Training, Human Factors, Clinical education

## Abstract

**Background:**

Laryngectomy (removal of the larynx, usually due to cancer) results in significant anatomical changes requiring specific clinical skills to safely manage the airway and support altered communication. It is crucial that healthcare professionals understand how to support people with laryngectomy, particularly in emergency care when their usual healthcare teams will not be present. Provision of laryngectomy training is limited. Existing education approaches fail to fully meet the needs of healthcare professionals, which in turn impacts on the provision of intervention to people with laryngectomy. With increasing evidence for simulation in pedagogical literature, this study explores how this approach can be used to support clinical skill education and improve urgent laryngectomy care. The aim of this study was to establish if a simulation-based approach is a feasible method of enhancing healthcare professional knowledge and confidence to provide emergency care to people with laryngectomy.

**Methods:**

A simulation-based training programme was piloted with delegates from a range of healthcare professions, over three separate study days. Immersive simulation scenarios were facilitated within a medical simulation centre using a modified SimMan mannequin, specially created models and prosthetics. Post-simulation debriefings were held with a focus on developing clinical skills within a Human Factors approach. In addition, training incorporated a skills-based session and interactive discussion with expert patients. Training was evaluated using pre- and post-course self-evaluation and qualitative feedback. Feasibility outcomes included the percentage of eligible participants who consented to take part, and the number of participants who completed the training.

**Results:**

Twenty-eight multidisciplinary healthcare professionals registered for the training; 26 (93%) attended and completed the training activities as prescribed. Qualitative data indicated that simulation, debrief and skills practice were all perceived as important training aspects. Participants placed particular value on the simulated resuscitation scenario. Self-assessed composite knowledge scores and individual knowledge-item scores increased significantly post-training (*p* =  < 0.001—0.04). Reflective of participants’ emphasis on resuscitation, knowledge of post-laryngectomy resuscitation requirements increased significantly post-training (*p* =  < 0.001).

**Conclusions:**

Simulation-based training is a feasible method of clinical skill acquisition for urgent laryngectomy care. Further research is needed to assess whether competence is maintained over time, and whether Human Factors learning generalises to clinical practice. Wider study could incorporate assessment of the impact of the training on people with laryngectomy's experiences of urgent care and potential impact on hospital flow.

**Supplementary Information:**

The online version contains supplementary material available at 10.1186/s12909-025-06964-8.

## Background

This study evaluates a novel simulation-based training for urgent laryngectomy care. Total laryngectomy is the permanent, surgical removal of the larynx, most commonly used to treat advanced throat cancer [1]. This results in separation of the respiratory and digestive tracts, with the upper airway refashioned to end at an anterior tracheostoma (front of neck airway).

The anatomical changes post-laryngectomy permanently alter respiratory function, with airflow for breathing permitted solely via the tracheostoma. This differs from tracheostomy, where the upper airway remains present, thus the tracheostomy airway can be temporary and reversible. Understanding this distinction is crucial; clinicians must therefore acquire the knowledge that people with laryngectomy (PWL) lack an upper airway whereas, in tracheostomised patients the upper airway remains in situ. Suboptimal knowledge or errors may result in significant safety events including delays in care, incorrect administration of oxygen to the nose or mouth [1] or critical events such as oral intubation of a neck breather resulting in harm or death [2].

Following laryngectomy, daily stoma care is required to maintain airway patency, as obstruction of the tracheostoma can lead to respiratory arrest. Some PWL wear a soft silicone tube to prevent shrinkage and ensure a sufficiently open stoma. Furthermore, the functions of the upper airway in warming, filtering and humidifying air before it reaches the lungs cease after laryngectomy. These functions are essential in preventing excess mucus build up and maintaining pulmonary health [3]. The lost upper airway functions must be replaced through use of an external device, known as a Heat, Moisture Exchange system (HME). This typically takes the form of an adhesive baseplate, which acts as housing for a replaceable cassette containing a material that provides a surface for condensation and absorption. Regular use of an HME can reduce the likelihood of mucus-related airway obstruction and associated impacts of upper airway loss (such as excessive coughing and tracheal irritation), [4]. Whilst an HME is the optimal approach to restoring humidification [5] not all PWL use HMEs. Instead, PWL may use alternatives such as humidification bibs, however the lesser restoration of humidity with these may introduce higher risk of respiratory difficulties which require medical attention.

With the removal of the larynx, the ability to produce voice is lost. An alternative method is therefore required to re-establish communication. Options for communication rehabilitation include use of an artificial larynx, oesophageal speech or surgical voice restoration. Where accessible, surgical voice restoration is the preferred method of restoring communication after laryngectomy [6–8]. A voice prosthesis (a small silicon device, also known as a valve) is placed between the trachea and oesophagus in a surgically created tract. This enables air to flow from the trachea into the reconstructed pharynx, eliciting vibration of the pharyngoesophageal tissues on exhalation [6]. The vibration produces an alternative source of phonatory sound, in the absence of the vocal folds, which is then shaped into speech by the articulators.

Following total laryngectomy, rehabilitation is essential to maintain a safe airway for all PWL, and to manage the voice prosthesis on an ongoing basis for those who have undergone surgical voice restoration [9]. Healthcare services are required to establish protocols for urgent laryngectomy care to resolve problems requiring swift medical attention [10]. This includes managing tracheostoma shrinkage or obstruction, which can lead to respiratory arrest; voice prosthesis failure, which can result in aspiration of food, fluid or saliva; or prosthesis displacement, which risks prosthesis-related airway obstruction, frank aspiration, loss of the tract for voicing, and requires immediate action to detect and remove the foreign body [11, 12]. Ongoing aspiration of food or fluids can also result in significant health complications, including respiratory distress or aspiration pneumonia, in addition to causing discomfort and disruption to PWL [13].

Post-laryngectomy rehabilitation is complex and is typically undertaken by Speech and Language Therapists/ Pathologists (SLTs / SLPs) with specialist training in head and neck cancer rehabilitation. National guidelines recommend that specialist SLTs are present in all head and neck cancer units [10]. There is, however, an insufficient number of head and neck cancer specialising-SLTs within UK practice, and this is recognised as an area of shortage within the SLT profession [14]. Professional guidelines outline the required knowledge and skills for head and neck cancer rehabilitation [15], yet pre-registration programmes provide only foundation training. Existing surveys of UK SLTs, North American and Australian SLPs demonstrate that current training approaches could be enhanced to better support practice. SLPs reported feeling unprepared to work with voice prosthesis users following pre-registration training [16] and highlighted the paucity of advanced-level training [17, 18]. Australian SLPs stated a desire for practical training that incorporates hands-on practice of clinical skills, shared reflection and learning, and avoidance of training in isolation [19].

Whilst SLTs typically support post-laryngectomy care needs within standard working hours, SLT services are rarely funded to provide evening and weekend cover. A challenge therefore arises for urgent laryngectomy care at weekends and evenings when healthcare professionals (HCPs) with limited or no knowledge of laryngectomy may be required to provide care. Out of hours laryngectomy care pathways are not standardised across the UK and the education of multidisciplinary HCPs is required [20]. Typically, PWL are advised to attend Accident and Emergency (A&E) departments if they have urgent care needs outside of SLT service hours, which has implications for demand on frontline services and hospital flow. This is pertinent in the context of an 18% increase in A&E attendances in the past decade [21]. The current model of laryngectomy care therefore presents two training needs. Firstly, head and neck cancer-specialising SLTs are a shortage profession with ongoing advanced training needs; and secondly, clinical upskilling is required for multidisciplinary HCPs who may be called upon to provide urgent laryngectomy care out of hours.

Within the healthcare setting, communities of practice [22] and apprenticeship learning [23] are established approaches to clinical learning [24]. In such approaches, an experienced clinician imparts knowledge to the trainee through direct participation, observation, supervised side-by-side practice and clinical skills sessions. However, PWL are a relatively small clinical population, therefore it may be challenging for the HCP to gain sufficient exposure to develop clinical reasoning and manual skills, and particularly to do so safely in pressured situations, such as those requiring urgent clinical intervention. The “learning through doing” approach also poses a dilemma in terms of maintaining the comfort, safety and well-being of PWL, particularly when procedures are invasive, high-risk or occur less frequently, as within the laryngectomy population. Furthermore, the availability of experienced SLTs who can provide an advanced level of laryngectomy care training may be insufficient due to workforce shortages. Hence, there is a need to develop alternative models of training for urgent laryngectomy care.

We propose simulation-based training as an adjunct to traditional learning methods. High-fidelity simulation is a powerful tool in skill and competency acquisition, with existing evidence across varied medical specialisms, including emergency medicine [25], geriatrics [26], gastroenterology [27] and cardio-thoracic surgery [28]. Simulation replaces real clinical experiences with guided scenarios which address gaps in knowledge, exposure and clinical skill, without reliance on patients as training resources [29]. Suited to interdisciplinary learning, simulation allows delegates to master technical skills (such as insertion of a laryngectomy tube, or tracheostoma cleaning) and non-technical skills (such as decision-making, communication, teamworking and situational awareness) in a low-risk environment. This permits educators to focus on specific learning tasks, without needing to balance teaching and clinical roles in a "live” clinical environment [30].

Consideration of non-technical skills in simulation provides the opportunity to consider Human Factors. The term Human Factors refers to the understanding of how people interact with each other, their environments and associated systems, with the aim of improving the outcomes and well-being of those involved [31]. HCPs are often required to make difficult decisions in dynamic, intense circumstances. Increases in the complexity and pressure of a situation may compromise decision-making, with a resultant impact on clinical outcomes, care quality and safety [32]. Given that urgent laryngectomy scenarios may occur in pressured clinical environments where multidisciplinary healthcare professionals could be in attendance (e.g. A&E, hospital wards), it is pertinent to use an interdisciplinary training, inclusive of Human Factors education, incorporating interpersonal and crisis resource management skills; analysis of interaction styles and cognitive skills; and decision-making, which are key to minimising risk of adverse events in healthcare [33].

Simulation-based learning is centred on sound educational principles, ensuring deep learning through active engagement in immersive scenarios, designed to accurately reflect the clinical environment [23, 34]. To enhance fidelity and provide realistic tactile, auditory and visual stimuli, simulation often involves use of trained actors, interactive mannequins and monitoring equipment [35]. Trainees engage in problem-centred, experiential learning, relevant to areas of responsibility [23]. Skill-development is achieved through clear instruction, honest self-reflection, constructive feedback and error correction to improve future performance. The reflective component helps trainees to internalise and generalise new skills and knowledge [36]. Healthcare simulations generally follow a standard pattern [37] as set out in Table 1.

**Table 1:** Elements of simulation training in healthcare (adapted from [37])

Element	Content
Pre-brief	Introduction to the simulation environment, technology and physical resources to be used in the scenarios Establishment of rules and expectations within the simulation environment Promotion of psychological wellbeing Introduction to principles of human factors and crisis resource management
Brief	Discussion between educational team and simulation team members to establish roles and intended learning outcomes Provision of basic information to set the scene for delegates
Simulation	Scenario takes place within simulation environment
Debrief	Delegates’ immediate reaction and emotional response to the scenario Analysis and reflective discussion, guided by trained facilitator Feedback and summary to highlight key learning points

Within this study, a novel simulation-based training for urgent laryngectomy care was designed and evaluated, to meet the following aims:

- To evaluate the feasibility of simulation-based training for urgent laryngectomy care
- To increase the knowledge and confidence of HCPs in managing urgent laryngectomy care needs
- To gain qualitative feedback on trainees’ experiences of the training

## Methods

### Study design and setting

Three training days were held as part of an existing ongoing laryngectomy training provision for multidisciplinary HCPs. However, the content of the training days was revised to include simulation and practical skills sessions, adding experiential learning to what was previously a didactic presentation training format. The training took place in the medical simulation suite of an NHS London teaching hospital.

### Participants

Participants were multidisciplinary HCPs employed by the hospital trust. They were eligible to attend the training if they worked with PWL routinely or worked in an area where they may encounter PWL out of hours, for example A&E staff or on-call Respiratory Physiotherapists.

### Sample size

Each training day was designed for nine participants to ensure fidelity in the simulation scenarios and to support shared, skills-based learning. This gave a total target of 27 participants over the three training days. The training was oversubscribed, and 28 HCPs were accepted to protect against non-attendance. The breakdown of attendees by profession is depicted in Table 2.

**Table 2:** Elements of the training and learning type

Learning activity in order of delivery (duration)	Description	Learning type
Theoretical lecture on laryngectomy care (60 min)	Didactic session covering anatomical and physiological changes post-laryngectomy	Acquisition Discussion
Introduction to Human Factors and crisis resource management (45 min)	Explanation of HF referencing high profile cases of clinical situations with poor outcomes because of human error. Introduction to principles of CRM, signposting to use in simulation scenarios	Acquisition Discussion
Simulation scenarios (× 4) (120 min total)	Immersive clinical scenarios based on urgent laryngectomy care needs	Practice Collaboration
Debriefing (× 4) (60 min total)	Post-simulation debriefing using HF-approach to create safe environment for group reflection and learning	Discussion
Skills-based session (120 min)	Small group practice of manual skills with facilitation from experienced SLTs Exploration of equipment, care needs and techniques	Practice Collaboration Discussion Production
Expert patient (45 min)	Question and answer session with expert PWL, with experienced SLT to facilitate	Discussion Investigation

### Training protocol

A novel simulation-based training was developed, which incorporated both didactic and practical learning. Taught elements included anatomical and physiological changes after laryngectomy; differentiation from tracheostomy; basic elements of laryngectomy care, such as stoma monitoring and the benefits of humidification and filtration devices (HMEs) in pulmonary rehabilitation; and the introduction of Human Factors and crisis resource management skills. Practical sessions involved immersive simulation scenarios; debriefing; clinical skills-based sessions, such as practicing stoma care or cleaning a voice prosthesis; and discussion with an expert PWL patient using tracheoesophageal speech and artificial larynx speech. The training was facilitated by experienced head and neck cancer-specialising SLTs supported by medical simulation centre staff. Table 3 describes the elements of the training and the associated learning type [38].

**Table 3:** Example simulation scenario

Element	Scenario content
Pre-Brief	Trainees introduced to simulation room Revision of Human Factors and crisis resource management principles Promotion of safe space for learning
Brief	**Trainee briefing:** You are reviewing a 58 year old male PWL who has been admitted with a new left sided embolic stroke. This affects strength and coordination of his hand. He has a voice prosthesis in situ but is struggling to use it **Trainer briefing:** One trainer required to act as Nurse and provide observations showing deterioration. One trainer required to act as voice of patient. As scenario progresses patient reports with increasing frequency that he feels unwell, hot and has indigestion. Patient then stops responding Once trainees commence cardiopulmonary rehabilitation (CPR), trainers to take the role of the resuscitation team, enter the scenario and support with ongoing CPR. Simulation ends
Simulation	**Clinical scenario:** Cardiac arrest in PWL SimMan mannequin with voice provided by trainer **Scenario plot:** As scenario progresses the patient starts to feel unwell, reporting that he is lightheaded, hot and has a feeling of indigestion. He then stops responding and goes into cardiac arrest **Learning outcomes and crisis resource management points (technical):** Recognising the deteriorating patient Resuscitation of a neck breather with altered upper airway Knowledge of resuscitation post-laryngectomy **Learning outcomes and crisis resource management points (non-technical):** Communicate effectively Anticipate and plan Re-evaluate repeatedly Use all available information Leadership and followership Call for help early—Cardiac arrest call is made Use SBAR (Situation, Background, Assessment, Recommendation) tool
Debrief	Discussion of trainees’ immediate reaction and emotional responses to scenario Analysis and reflective discussion in group to promote shared learning Feedback and summary of key learning points **Debrief prompts:** Were Human Factors and crisis resource management elements used successfully? What were the differences between this scenario and other resuscitation situations? Did the differences affect your confidence in managing this scenario?

### Simulation scenarios

Four clinical scenarios were devised for the central immersive simulations. The scenarios were based upon common urgent presentations and incorporated [1] a blocked or shrinking tracheostoma (including foreign body blockage), requiring delegates to identify the risk, make appropriate use of suction and nebuliser devices and successfully stent the stoma; [2] dislodgement of the voice prosthesis causing frank aspiration of saliva, necessitating risk assessment, use of equipment to appropriately manage leak, demonstration of understanding of potential foreign body management; [3] dislodgement of the voice prosthesis causing closure of the tracheoesophageal tract, requiring identification of the problem and assessment of the tract; and [4] cardiac arrest requiring resuscitation via the tracheostoma. Table 4 provides a full description of the simulation scenario for cardiac arrest, incorporating the elements of simulation training in healthcare [37].

**Table 4:** HCP trainees accepted to training

Profession	Number of trainees accepted (*n* = 28)	Percentage of trainees
Nurse	9	32%
Physiotherapist	7	25%
Ear, Nose and Throat (ENT) doctor or surgeon	7	25%
SLT	5	18%

### Training materials

**Fig. 1:**
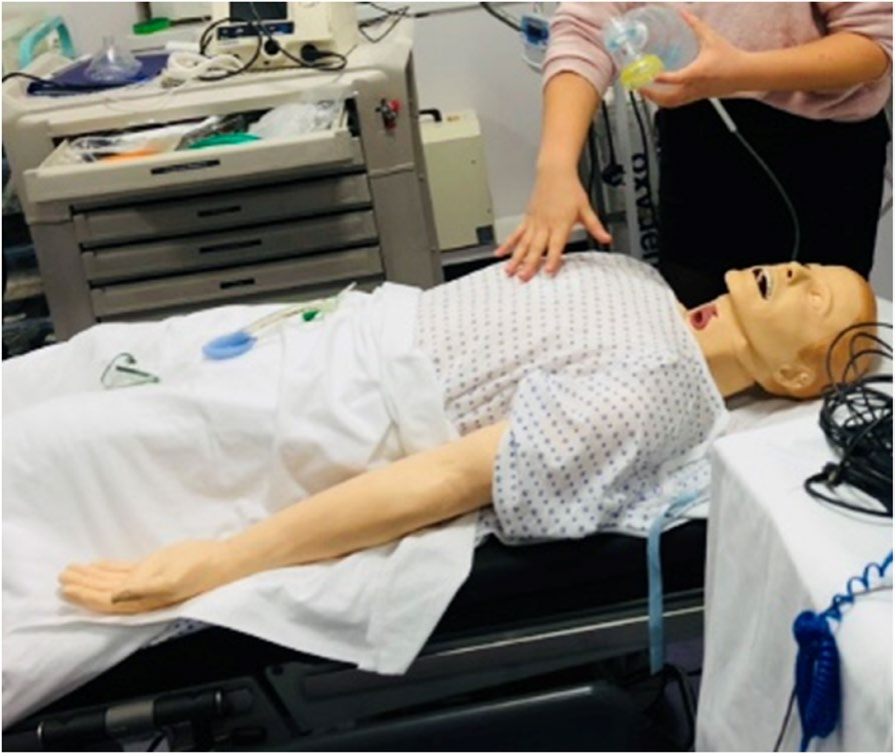
SimMan mannequin

**Fig. 2:**
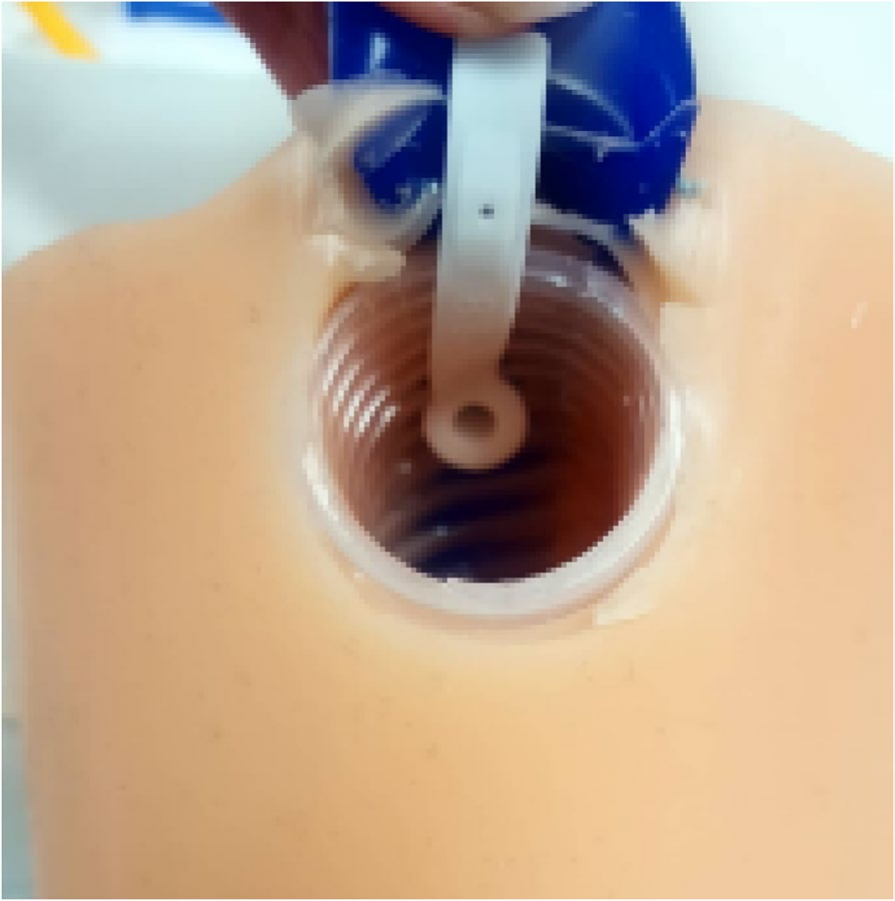
Wearable prosthetic with voice prosthesis

Training took place in a high-fidelity medical simulation room with separate classroom for debriefing, theoretical and skills-based sessions. A SimMan mannequin with front of neck airway was used for the resuscitation and tracheostoma shrinkage scenarios (Fig. 1). Medical simulation trained actors or experienced HNC-specialising SLTs played the role of a PWL in the remaining scenarios. Prosthetics were created by the simulation centre staff and worn by actors or SLT trainers to present the appearance of a tracheostoma and voice prosthesis (Fig. 2). The simulation room controls were used to display dynamic vital signs of the patient and to communicate (as the patient) to trainees during the immersive scenario. Skills-based practical sessions incorporated the use of common laryngectomy equipment such as voice protheses, stoma protectors, forceps and stoma care items. Prosthetic models of the tracheostoma and trachea were created to enable trainees to practice insertion and care of a voice prosthesis.

### Measures

Participants completed the following evaluation measures before and after the training:

- Self-assessment of knowledge and confidence in the management of urgent laryngectomy care, using an eight-item questionnaire, scored with a five-point Likert scale rated from one – five per item (Additional File 1). Higher scores indicated increased knowledge or confidence. SLTs and ear, nose and throat (ENT) doctor participants answered an additional five questions pertaining to their role in voice prosthesis management.
- Qualitative questionnaire capturing reflections on the training (Additional File 2)

In addition to the above evaluation measures, feasibility was evaluated using the following outcomes:

- Number of HCPs signing up to the training
- Analysis of trainees by profession
- Proportion of HCPs who complete the learning activities of the training day

### Data analysis

Descriptive statistics were used to report participant profession and feasibility outcomes. Wilcoxon signed-ranks tests were used to analyse pre- and post-course self-assessment. Effect sizes were calculated (*r* = *z / √n*) and interpreted as r < 0.1 no effect / very small effect; r = 0.1 small effect; r = 0.3 medium effect, r = 0.5 large effect [39]. Statistical analysis was carried out using SPSS version 29.0.2.0. Qualitative data was analysed to identify the key themes arising in trainee feedback. The DoCTRINE guidelines were used to inform reporting of the study [40].

## Results

### Participant characteristics

In total, 28 multidisciplinary HCPs were accepted to the training, the majority of whom were Nurses (*n* = 9, 32%). Table 1 above provides the breakdown of trainees by profession.

### Feasibility

Recruitment activity generated positive interest in the training and the recruitment target was exceeded. HCPs requesting training after capacity was reached were offered a waiting list place for future training days. All HCPs who applied for the training were eligible to attend. A range of multidisciplinary HCPs attended the training, however no HCPs from A&E departments applied to attend. Two HCPs did not attend the training (ENT doctor, due to clinical emergency *n* = 1; Nurse, unknown reason *n* = 1). The remaining HCPs (*n* = 26, 93%) attended the full training day and completed all learning activities.

### Qualitative data

The training was rated as excellent by 96% of trainees (*n* = 25), with one non-response. Overall, delegates reported that simulation was an effective method of addressing the practical aspects of laryngectomy care. Of the trainees, 69% (*n* = 18) perceived the simulation scenarios and debrief to be the most useful aspects of the training, with the resuscitation scenario being particularly valued. The importance of the taught session was highlighted (*n* = 7) for introducing or refreshing knowledge of anatomical changes post-laryngectomy. One delegate described the inclusion of the expert patient as the most beneficial element to their learning. Table 5 depicts the main themes generated from the qualitative feedback.

**Table 5:** Themes with illustrative examples of most valued aspects of the course, as described by trainees

Theme	Trainee feedback
Theme 1: Benefits of simulation scenarios	“Getting to interact with a laryngectomy patient.” “Practical scenarios with real patient.” “Watching and participating in scenarios.” “Scenarios I have not encountered before.”
Theme 2: Technical skill practice	“Having a large practical element to the day was really helpful.” “Seeing and feeling equipment and practicing inserting [valves].” “Practice inserting small gel caps and valve change.” “Hands-on experience.”
Theme 3: Educator delivery	“Very engaging and interactive, supportive leaders.” “Time for questions and discussion.” “Trainers knew what they were talking about and very helpful in answering all questions.” “Non-judgemental feel, able to ask all of my questions. Very approachable team.”
Theme 4: Transference to clinical practice	“There were really useful tips for day to day practice.” “Excellent training which has made me feel confident in managing basics of patients’ laryngectomy and what to do in an emergency situation.” “The practical session is very useful. I feel more confident in dealing with laryngectomy in terms of dealing with an emergency.” “It was all very useful for me, I had little knowledge of laryngectomy before and I now feel a lot more comfortable.”

There was full consensus from participants that all elements of the training were enjoyable and useful. Suggestions for improvement were minimal, but included ideas for supplementary content, for example adding nursing perspectives (*n* = 1), discussion of emergency algorithms (*n* = 1), inclusion of suction techniques (*n* = 1) and scenarios differentiating approaches for laryngectomy and tracheostomy (*n* = 1). The need for increased exposure and ongoing opportunity for practice was also highlighted by one participant:

*“Where I’m not confident is no reflection on trainers / training but my own need for exposure.”*

These views were isolated comments from individual participants and lacked sufficient frequency and consensus to be considered themes in their own right. Nevertheless, the information is useful for planning future training.

### Self-assessment

Wilcoxon signed-rank tests showed significant increase in self-assessment scores across all individual items, and in composite knowledge score (All participants—*p* = 0.04; ENT and SLT additional items—*p* = 0.005), as illustrated in Table 6. Median composite scores for self-assessed confidence improved post-training, however this did not reach significance (All participants—*p* = 0.10; ENT and SLT additional items—*p* = 0.06).

**Table 6:** Pre and post training self-assessment scores

Question	Pre-score Median (IQR)	Post-score Median (IQR)	Z	*P* value	Effect size
**All participants:**					
I can state the changes in anatomy following laryngectomy	4.00 (3.00–4.00)	4.75 (4.00–5.00)	−3.48	< 0.001	0.70
I can state the changes in breathing function following laryngectomy	4.00 (3.75 – 4.00)	4.75 (4.00–5.00)	−3.77	< 0.001	0.75
I can state the changes in swallowing function following laryngectomy	3.80 (2.75–4.00)	4.75 (4.00–5.00)	−3.17	0.002	0.63
I am aware of the differences in resuscitation post-laryngectomy	3.20 (3.00–4.00)	4.60 (4.00–5.00)	−4.14	< 0.001	0.83
I know what a voice prosthesis looks like	4.00 (3.00–4.10)	4.88 (4.75–5.00)	−3.65	< 0.001	0.73
Median for knowledge items (all participants)	4.00 (3.50 – 4.00)	4.75 (4.67 – 4.81)	−2.03	0.04	0.40
I feel confident assessing the appearance and patency of a laryngectomy stoma	3.60 (2.75–4.00)	3.75 (4.00–5.00)	−3.50	< 0.001	0.70
I feel confident to carry out daily stoma care	3.50 (2.00–4.00)	4.25 (4.00–5.00)	−3.23	< 0.001	0.64
I feel confident in managing emergency situations with laryngectomy patients	2.80 (2.00–3.00)	4.25 (4.00–4.44)	−4.16	< 0.001	0.83
Median for confidence items (all participants)	3.50 (2.80 – 3.60)	4.25 (3.75 – 4.25)	−1.60	0.10	0.32
**ENT and SLT participants only:**					
I feel confident in carrying out a straightforward prosthesis change	3.20 (3.20–3.20)	4.70 (4.00–4.70)	−2.75	0.006	0.55
I feel confident in managing central leak	2.60 (2.60–3.00)	4.30 (4.00–4.30)	−2.81	0.005	0.56
I feel confident in managing peripheral leak	2.40 (2.40–3.00)	4.30 (4.00–4.30)	−2.91	0.004	0.58
I feel confident using prosthesis troubleshooting techniques	3.00 (3.00–3.00)	4.30 (4.00–4.30)	−2.81	0.005	0.56
Median for confidence items (ENT and SLT only)	2.80 (2.45 – 3.15)	4.30 (4.30 – 4.60)	−1.83	0.06	0.37
I understand the difference between voice prosthesis types	3.00 (3.00–3.00)	4.20 (4.00–4.20)	−2.81	0.005	0.56
Median for knowledge item (ENT and SLT only)	3.00 (3.00–3.00)	4.20 (4.00–4.20)	−2.81	0.005	0.56

## Discussion

This study evaluated the application of a novel simulation-based training for urgent laryngectomy care. The training was feasible for multidisciplinary HCPs and received positive feedback from trainees. Self-assessed knowledge relating to laryngectomy care was improved post-training across all domains. Self-assessed confidence scores improved post-training, however there was no significant difference between composite scores on pre- and post-training self-assessment. This points towards the need to consider how specific knowledge acquired during the training can be transferred to the clinical environment to increase overall confidence.

Whilst the training was oversubscribed and attended by a variety of HCP disciplines, no trainees attended from the A&E department. PWL are often directed to A&E departments for urgent out of hours care, therefore engagement of A&E department staff may be important in countering reported reduced confidence in skills in this clinical area [20], and improving the healthcare experience of PWL. Consequently, the engagement of A&E staff merits further attention to ensure that future training is accessible for A&E staff and communicates the relevance of the training to the A&E setting.

PWL have reported concern about potential negative resuscitation outcomes should they be assisted by an HCP uninformed in laryngectomy care during a respiratory crisis [41]. Notably, trainees gave especially positive feedback about the inclusion of a resuscitation scenario in the simulation training, demonstrating accord between the priorities of PWL and trainees. Consensus here may reflect the paucity of laryngectomy resuscitation training, even among those healthcare professions who are likely to encounter this population in their daily role, such as ENT medics and SLPs/SLTs.

Trainees reported that the simulation training was beneficial and supported learning. This is consistent with the use of simulation-based learning in other clinical fields where increased confidence and clinical skill acquisition have been demonstrated; such as tracheostomy care [42], telepractice [43] and nasendoscopy training [44].

Strengths of the training included targeted learning objectives to increase relevance to interdisciplinary trainees, and a consistent curriculum across the training days, which minimised variability of teaching. The multi-modal use of actors, prosthetics and mannequins led to high fidelity scenarios, accurately reflecting clinical environments and situations. Furthermore, teaching modalities combining taught, practical and expert patient content accounted for differing learning styles. The training team used simulation-specific debriefing techniques and interactive post-scenario debriefs to enable trainees to discuss experiences, reflect and learn from each other. Practical skills-based sessions were supported by experienced SLTs who provided immediate feedback and instruction to improve performance in technical procedures, with opportunity for repetitive practice to embed learning. Repetitive practice of a clinical skill using simulation models has been shown to increase experience and clinical confidence [44]. The inclusion of an expert patient provided context on the lived experience, however, their role could have been expanded to yield additional benefits, such as to support debriefing sessions from a patient perspective. Study of expert patient educators [45] has highlighted that peer-support and training could enhance the expert patient role in health education, whilst healthcare students report positive perceptions of patient educators [46].

Study limitations include a small sample size from one site, and the lack of control group to compare simulation-based learning outcomes against traditional training methods, however it should be borne in mind that the primary aim of this evaluation was to assess whether simulation-based learning was feasible and effective for urgent laryngectomy care training. Outcomes are based on self-rating of skills and confidence which may not reflect changes in practice, and self-perception may vary. Use of competency frameworks may support delegates and trainers to more accurately assess knowledge and skills and minimise potential variability in responses. It is of note that 25% of participants were ENT resident doctors. There is, therefore, the potential that existing baseline knowledge could have introduced bias into the study results. Future iterations of the training could seek to increase sample size to allow for analysis of results by healthcare profession.

Additional outcome measures analysing potential impact on flow through emergency departments, associated costs, and patient experience would enhance the evaluation of simulation-training efficacy and value. In addition, while this evaluation demonstrated the merits of simulation-based learning in technical skill and knowledge development, evaluation of Human Factors learning was lacking. Human Factors is complex and clinical experiences and interactions will vary widely between professional groups. However, the principles of situational awareness, communication, leadership and teamwork are key elements which apply to all situations and roles, and form part of everyday work in relation to patient care [47]. Inclusion of a method to highlight this learning in the context of laryngectomy management, such as use of reflection to identify and mitigate potential future errors within attendees’ own clinical areas or specialisms, may enrich future training outcomes.

Future developments should incorporate evaluation of how learning generalises to clinical practice; Human Factors outcomes; increasing confidence in practice; and whether simulation-based learning is effective in reducing skill-decay in comparison to traditional training methods. Retention of airway management skills has been shown to be optimised through regular practice and feedback [48]. Similarly, opportunities for further learning and training exposure are key factors for SLTs in maintaining confidence in voice prosthesis management [19]. Future programmes should therefore consider refresher sessions for skill maintenance. Simulation has been shown to be effective in partially replacing clinical training time for SLPs [49]. This may be relevant for HCP trainees outside of major teaching hospitals where there may be less exposure to experienced clinicians who can support in-practice training. The application of additional technologies such as augmented-reality also merits exploration as a potential training adjunct.

## Conclusion

The use of simulation-based training for urgent laryngectomy care was feasible, and preliminary efficacy data demonstrated improvements in HCP knowledge of managing urgent laryngectomy care. Simulation-training is not intended to replace in-post learning, however it may enhance traditional clinical skill training to support knowledge acquisition and skill development. Simulation-based training can support skill mastery through instruction, repetitive practice and integration of learnt curriculum into real-life scenarios, with immediate feedback and redirection where needed, yet without impact on patient experience. Future training should be based on learning principles and explore the potential of additional learning technologies such as augmented-reality training. Furthermore, importance must be given to maintenance of competence, particularly for clinicians with reduced clinical exposure to laryngectomy. Future research is required to evaluate Human Factors outcomes, transference to clinical practice and impact on patient experience.

## Supplementary Information

Additional file 1. Self-evaluation questionnaire.

Additional file 2. Qualitative questions.

## Data Availability

The datasets used and/or analysed during the current study are available from the corresponding author on reasonable request.

## Abbreviations

A&E: Accident and Emergency
HME: Heat moisture exchanger
HNC: Head and Neck Cancer
PWL: People/person with laryngectomy
SLP: Speech and Language Pathologist
SLT: Speech and Language Therapist
