# Supplementary material for: Evaluation of a novel simulation-based training for urgent laryngectomy care
Source: BMC Med Educ. 2025 Mar 26;25:442. doi: 10.1186/s12909-025-06964-8 (PMC11948698; doi:10.1186/s12909-025-06964-8)
Supplement: Supplementary file 1 — Additional file 1. Self-evaluation questionnaire. [file 12909_2025_6964_MOESM1_ESM.docx]

**Additional File 1: Self-evaluation questionnaire**

**Self-evaluation:**

**1: strongly disagree, 2: disagree, 3: unsure, 4: agree, 5: strongly agree**

**All participants:**

| Knowledge questions |  |
| --- | --- |
| I can state the changes in anatomy following laryngectomy | **1 2 3 4 5** |
| I can state the changes in breathing function following laryngectomy | **1 2 3 4 5** |
| I can state the changes in swallow function following laryngectomy | **1 2 3 4 5** |
| I am aware of the differences in resuscitation post-laryngectomy | **1 2 3 4 5** |
| I know what a voice prosthesis looks like | **1 2 3 4 5** |
| Confidence questions |  |
| I feel confident assessing the appearance and patency of a laryngectomy stoma | **1 2 3 4 5** |
| I feel confident carrying out daily stoma care | **1 2 3 4 5** |
| I feel confident in managing emergency situations with laryngectomy patients | **1 2 3 4 5** |

**For ENT and SLT participants only:**

| Knowledge questions |  |
| --- | --- |
| I understand the difference between voice prosthesis types | **1 2 3 4 5** |
| Confidence questions |  |
| I feel confident in carrying out a straightforward voice prosthesis change | **1 2 3 4 5** |
| I feel confident in managing central leak | **1 2 3 4 5** |
| I feel confident in managing peripheral leak | **1 2 3 4 5** |
| I feel confident using prosthesis troubleshooting techniques | **1 2 3 4 5** |
