# Supplementary material for: Evaluation of a novel simulation-based training for urgent laryngectomy care
Source: BMC Med Educ. 2025 Mar 26;25:442. doi: 10.1186/s12909-025-06964-8 (PMC11948698; doi:10.1186/s12909-025-06964-8)
Supplement: Supplementary file 2 — Additional file 2. Qualitative questions. [file 12909_2025_6964_MOESM2_ESM.docx]

**Additional File 2: Qualitative questions**

**Qualitative questionnaire**

Please rate this training overall:

**Excellent** **Good**  **Average** **Below average Poor**

**Which parts of the training were most useful to you?**

**Was there anything that you did not enjoy or did not find useful?**

**Was there anything else you would like to have been included in the training?**

**How did you feel about the immersive simulation scenarios?**

**How will this training affect your clinical practice?**

**Any other comments?**
